# Implicit modeling of narrow vein type ore bodies based on Boolean combination constraints

**DOI:** 10.1038/s41598-022-10005-5

**Published:** 2022-04-12

**Authors:** Deyun Zhong, Ju Zhang, Liguan Wang, Lin Bi

**Affiliations:** 1 School of Resources and Safety Engineering, Central South University, Changsha, 410083 China; 2 Research Center of Digital Mine, Central South University, Changsha Hunan, 410083 China

**Keywords:** Energy science and technology, Engineering, Mathematics and computing

## Abstract

In this paper, we implement an automatic modeling method for narrow vein type ore bodies based on Boolean combination constraints. Different from the direct interpolation approach, we construct the implicit functions of the hanging wall and foot wall surfaces, respectively. And then the combined implicit function is formed to represent the complete ore body model using the Boolean combination constraints. Finally, the complete ore body is obtained by Boolean operation of the hanging wall and foot wall surfaces. To model complex vein surfaces, some modeling rules are developed to allow the geological engineers to specify vein thickness constraints and vein boundary constraints. The method works for narrow vein type ore bodies (e.g., vein gold deposits and mineral sand deposits) which are large in two dimensions and narrow in the third. Taking the implicit function of radial basis functions interpolation as an example, several experiments are carried out by using the real geological sampling data of the mines. The experimental results show that the method is suitable for the modeling of narrow vein type ore bodies.

## Introduction

The implicit modeling method^1–4^ obtains the implicit function of the 3D geological body model by solving the interpolation equation satisfying the geological constraints, and then obtains the 3D surface mesh model by polygonizing the implicit function. Although most spatial interpolation methods can be applied to implicit modeling, considering the superiority of interpolation extrapolation and performance, the spatial interpolation methods based on kriging (e.g., universal cokriging^5^) and radial basis functions (e.g., RBF^6,7^, HRBF^8,9^ and GRBF^10,11^) are widely used in geological modeling.

At present, for the geological modeling with complex geometry shape features, the modeling effect of the existing implicit modeling methods often cannot satisfy the actual application requirements of mines. One of the most important problems is the lack of constraint rules of implicit modeling for different types of geological bodies. Although the 3D model can be controlled by adding more interpolation constraints, it will greatly affect the automation of the implicit modeling method. In addition, it is often difficult to construct the manual interpolation constraints in three-dimensional space.

The interpolation constraints constructed by sparse and uneven geological data are often difficult to represent the complex geological conditions, resulting in the significant difference between the implicit modeling results and the geological realistic expectations. It greatly limits the wide application of the implicit modeling software in actual mines. As a classic example, the modeling and grade estimation of vein type ore bodies with small thickness is an important challenge in practical application. The narrow vein type geological body has the characteristics of thin thickness and layered distribution, which can be regarded as a thin stratified model composed of hanging wall and foot wall surfaces, and its corresponding geological sampling data has obvious sparse and uneven characteristics. As the vein type ore bodies are complex and narrow in one dimension, it is difficult and time consuming to interpolate valid and faithful models by constructing manual interpolation constraints using the traditional interpolation methods^12–16^. Actually, in some cases, the 2.5D vein modeling is analogous to the coal seam modeling^17^. However, most of the existing modeling method of narrow vein type surfaces relies on two dimensional methods to estimate the thickness of the model.

In general, the implicit modeling methods control the geometry shape of the model by constructing interpolation constraints. However, for the narrow vein type geological bodies, if the traditional modeling method is used to construct interpolation constraints directly, the interpolation extrapolation result of the model may be quite different from the realistic shape of the model. The small thickness of narrow vein type geological bodies makes it very difficult for both the processes of implicit function interpolation and implicit surface reconstruction^18,19^. For the implicit function interpolation, if the hanging wall sampling data and foot wall sampling data are not interpolated separately, the interpolation result will be extremely poor. For the implicit surface reconstruction, considering that the average thickness of vein type ore bodies is generally far less than the surface reconstruction accuracy (the size of the cube). If the classical marching cubes methods^20–22^ are used to extract the isosurface directly, the reconstruction result may not recover the realistic geometry shape of the target implicit function. Therefore, in the process of implicit modeling for vein type ore bodies, the specific shape features should be considered to guide both the interpolation and reconstruction processes.

We try to construct modeling rules based on interpolation constraints to deal with the problems of geological modeling with special geometry shape features. From the perspective of model constraint effect, the interpolation constraints are used to control the local geometry shape features of the model, while the modeling rules are used to control the global geometry shape features of the model. For example, although the thickness of vein type ore bodies is very thin, this type of model can be regarded as the combination of a hanging wall surface (HW surface) and a foot wall surface (FW surface). Therefore, to avoid the potential issues of direct interpolation, a more realistic outcome will be achieved if the hanging wall and foot wall surfaces are interpolated separately and these interpolations are combined to form the vein model. It is worth noting that early researchers proposed that the mature commercial software (e.g., Leapfrog Geo, geological implicit modeling software) has integrated the vein surface modeling method based on the similar idea for a long time^23–25^, but the details of relevant researches have not been published for commercial reasons.

In this paper, we implement and verify the feasibility of vein surface modeling method based on the Boolean combination constraints, and further analyze and discuss the geological rule constraints in vein surface modeling. The method no longer constructs a single implicit function directly based on the geological sampling data. We construct the implicit function of hanging wall surface and the implicit function of foot wall surface respectively by distinguishing the hanging wall and foot wall sampling data. And then the combined implicit function is formed to represent the complete ore body model using the Boolean combination constraints. Moreover, for the reconstruction of implicit surface, we no longer extract the isosurface for the combined implicit function directly. On the contrary, we reconstruct the hanging wall surface and the foot wall surface using the corresponding implicit functions, respectively. Finally, the complete ore body is obtained by Boolean operation of the hanging wall and foot wall surfaces. The method works for narrow vein type ore bodies (e.g., vein gold deposits and mineral sand deposits) which are large in two dimensions and narrow in the third. Besides the interpolation constraints, some additional modeling rules should be developed to make the interpolated implicit function fits the unknown surface well. In this paper, we construct some modeling rules that allow the geological engineers to specify vein thickness constraints and vein boundary constraints. It is useful to model complex vein surfaces. For example, a minimum thickness can be specified to avoid potential surface mesh cross-overs. Based on the similar idea, more novel modeling rules can be developed to improve the reliability and efficiency of modeling results.

## Overview of the method

The potential field function $f\left({\varvec{x}}\right)$ is used to represent the mineralization domain of the ore body model, when the implicit modeling method is used to model the ore body. The common method is to regard the mineralization domain where the ore body model is located as a signed implicit function field. The implicit function field represented by Euclidean distance is the classical signed distance field (SDF)^16,19^. Among them, the sign of implicit function values represents the internal and external position relationships of the ore body models in the mineralization domain. Without loss of generality, we agree that the function values of the points outside the orebody are positive and the function values of the points inside the orebody are negative. Note that this definition of the sign of function values is different from the convention used by Cowan et al.^23^.

According to the above definition, the relationship between the mineralization domain of ore body model and the sign of implicit function $f({\varvec{x}})$ can be expressed as

1 $$f\left({\varvec{x}}\right)=sdist\left({\varvec{x}},{{\varvec{x}}}^{\boldsymbol{^{\prime}}}\right)=\left\{\begin{array}{l}=0, when \, x \, is \, on \, the \, surface \, of \, orebody \, model \\ > 0,\, when\, x\, is\, outside \,the\, surface \,of \,orebody\, model\\ < 0, \,when\, x \,is \,inside \,the\, surface\, of\, orebody\, model\end{array}\right.$$

where ${\varvec{x}}$ is any point in the mineralization domain, ${\varvec{x}}{^{\prime}}$ is the nearest point of ${\varvec{x}}$ on the implicit surface ${\varvec{S}}$ and $sdist({\varvec{x}}, {\varvec{x}}{^{\prime}})$ is the signed distance between ${\varvec{x}}$ and ${\varvec{x}}{^{\prime}}$.

Several types of surfaces are defined in the process of modeling vein surfaces, including the hanging wall surface, the foot wall surface and the mean surface. The hanging wall surface (HW surface) is used to represent the surface on the upper side of the vein object, which is always above the mean surface. The foot wall surface (FW surface) is used to represent the surface on the lower side of the vein object, which is always below the mean surface. The hanging wall and foot wall surfaces make up the whole model of vein object. The mean surface is an intermediate surface which is obtained by interpolating the geological sampling data of the hanging wall and foot wall surfaces. It is used as a reference surface of the hanging wall and foot wall surfaces to construct combination constraints.

**Figure 1:**
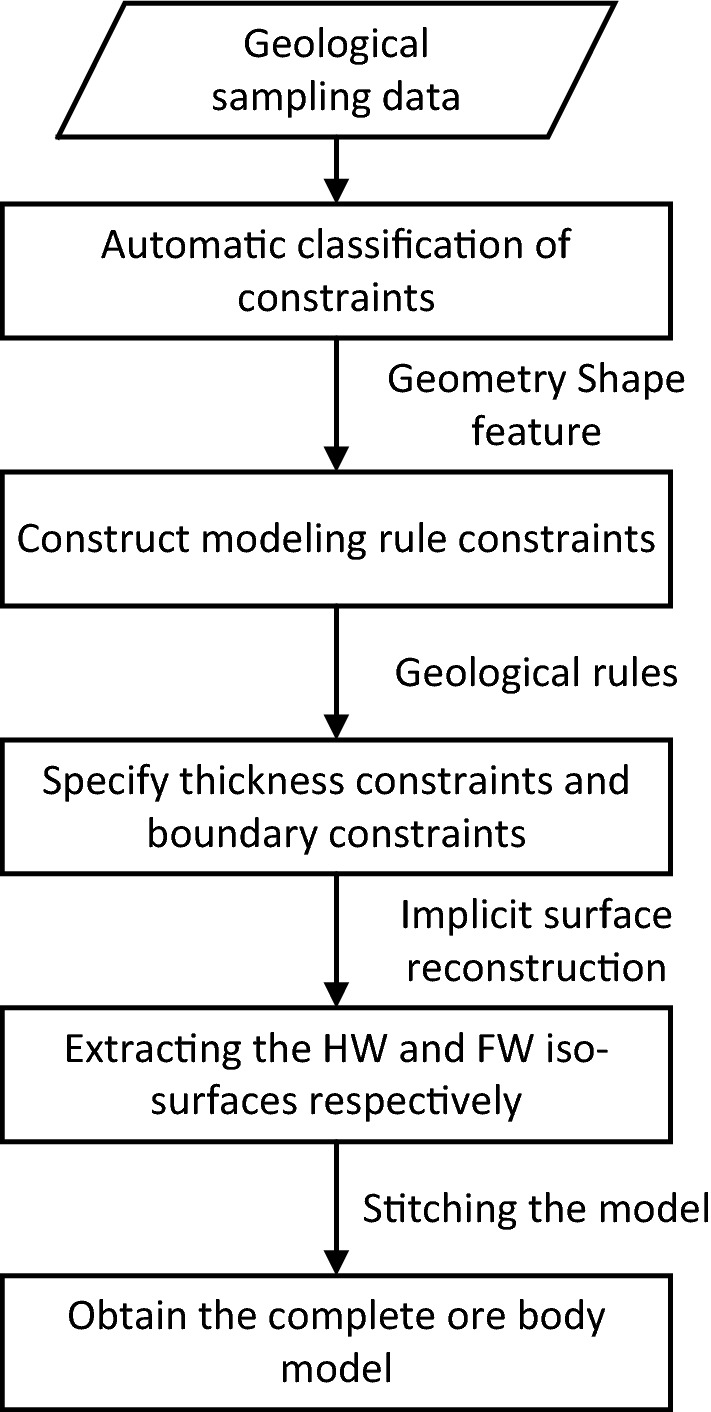
Overall flow chart of the vein surface modeling method.

The basic idea of the vein surface modeling is to combine the hanging wall implicit function and the foot wall implicit function based on Boolean combination constraints^26^. In this paper, the basic strategy of automatic modeling method for narrow vein ore bodies is to sample (geological constraints), interpolate (implicit function) and reconstruct (mesh model) the hanging wall surface and the foot wall surface of the vein object, respectively. The method is mainly composed of the following steps, and the overall flow chart is shown in Fig. 1.

**Step 1**: According to the geometry shape features of geological sampling data, construct the sampling points of the hanging wall surface and the sampling points of the foot wall surface, respectively.

**Step 2**: The implicit modeling method based on radial basis function interpolation is used to obtain the interpolation constraints of the hanging wall and foot wall surfaces.

**Step 3**: By solving the interpolation equations separately, the hanging wall implicit function and the foot wall implicit function are obtained. The implicit functions will be combined to construct modeling rules satisfying geometry shape features of vein object.

**Step 4**: According to the Boolean combination constraints based on the signed distance field, the vein thickness constraints and the vein boundary constraints can be constructed, so that the geological engineers can adjust the modeling results according to the prior geological rules.

**Step 5**: The implicit surface reconstruction method is used to extract the hanging wall isosurface and the foot wall isosurface, respectively.

**Step 6**: After combining the hanging wall and foot wall surfaces using the polygon Boolean operation method, the complete ore body model is obtained.

## Vein surface modeling

### Boolean combination constraints

The combination constraints of implicit function fields represent the constraints constructed by combining the implicit function fields according to the idea of Boolean combination operations between fields. Based on the idea of signed distance field, the combination of implicit functions can be regarded as the combination of signed distance fields. Taking two signed distance implicit functions ${f}_{A}\left({\varvec{x}}\right)$ and ${f}_{B}\left({\varvec{x}}\right)$ as an example, the combination constraints of implicit function fields can be divided into the following four types: intersection operation, union operation, complement operation and difference operation.

1. The combined implicit function $F\left({\varvec{x}}\right)$ constructed by the intersection operation of ${f}_{A}\left({\varvec{x}}\right)$ and ${f}_{B}\left({\varvec{x}}\right)$ can be represented as
  2 $$F\left({\varvec{x}}\right)={f}_{A}\left({\varvec{x}}\right)\cap {f}_{B}\left({\varvec{x}}\right)=max\left({f}_{A}\left({\varvec{x}}\right),{f}_{B}\left({\varvec{x}}\right)\right)$$
2. The combined implicit function $F\left({\varvec{x}}\right)$ constructed by the union operation of ${f}_{A}\left({\varvec{x}}\right)$ and ${f}_{B}\left({\varvec{x}}\right)$ can be represented as
  3 $$F\left({\varvec{x}}\right)={f}_{A}\left({\varvec{x}}\right)\cup {f}_{B}\left({\varvec{x}}\right)=min\left({f}_{A}\left({\varvec{x}}\right),{f}_{B}\left({\varvec{x}}\right)\right)$$
3. The combined implicit function $F\left({\varvec{x}}\right)$ constructed by the complement operation of ${f}_{A}\left({\varvec{x}}\right)$ can be represented as
  4 $$F\left({\varvec{x}}\right)=-{f}_{A}\left({\varvec{x}}\right)$$
4. The combined implicit function $F\left({\varvec{x}}\right)$ constructed by the difference operation of ${f}_{A}\left({\varvec{x}}\right)$ and ${f}_{B}\left({\varvec{x}}\right)$ can be represented as
  5 $$F\left({\varvec{x}}\right)={f}_{A}\left({\varvec{x}}\right)\ominus{f}_{B}\left({\varvec{x}}\right)=max\left({f}_{A}\left({\varvec{x}}\right),-{f}_{B}\left({\varvec{x}}\right)\right)$$

### Implicit function interpolation

#### Interpolation constraints

To obtain the implicit functions representing the hanging wall and foot wall surfaces, it is necessary to obtain the sampling data of the hanging wall and foot wall. According to the geometry shape features of geological sampling data, extract the hanging wall sampling point set ${{\varvec{P}}}_{1}$ and the foot wall sampling point set ${{\varvec{P}}}_{2}$, respectively. And the users should be allowed to adjust the sampling points to avoid unexpected errors.

**Figure 2:**
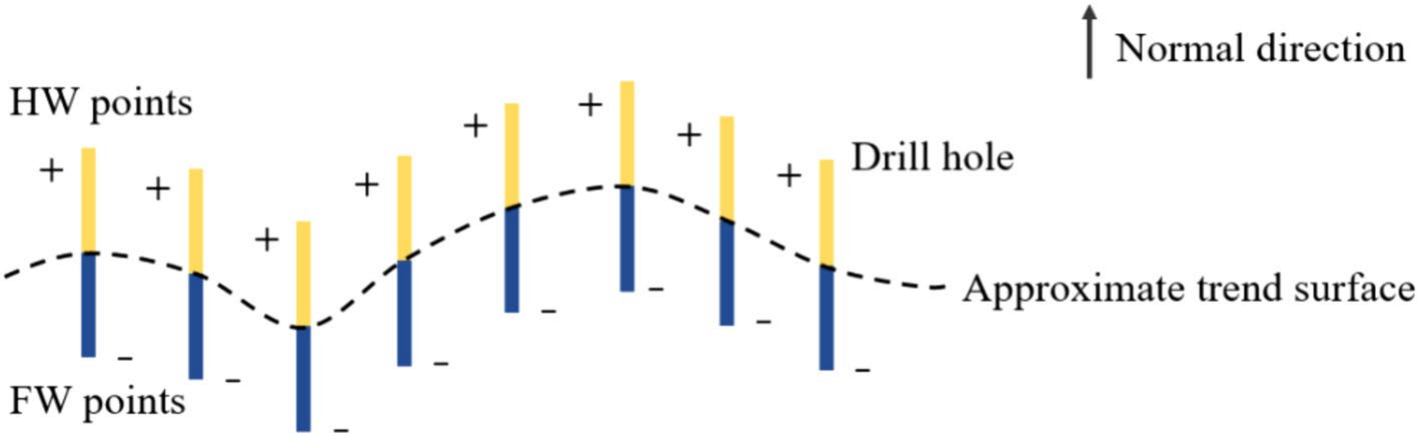
Construct interpolation constraints based on the drillhole data.

Taking the drillhole data as an example, to extract the sampling points of hanging wall and foot wall respectively, the approximate trend surface should be fitted according to all the sampling points. And then the sampling points are divided into hanging wall and foot wall points roughly according the signed distance values corresponding to the approximate trend surface. The signed distance values of hanging wall points are positive and the signed distance values of foot wall points are negative corresponding to the approximate trend surface, as shown in Fig. 2. Finally, the abnormal points are adjusted manually. In some cases, it is necessary to select the hanging wall and foot wall contact points manually for the complex ore deposits. A simple approach is to reverse the orientations of the drillhole segments directly.

Considering the superior extrapolation of the RBF interpolation method, the implicit modeling method based on RBF interpolation is used to convert the geological sampling data into interpolation constraints through the idea of signed distance field. Firstly, the sampling points in ${{\varvec{P}}}_{1}$ are used to construct the hanging wall interpolation constraints ${{\varvec{C}}}_{1}$ and the sampling points in ${{\varvec{P}}}_{2}$ are used to construct the foot wall interpolation constraints ${{\varvec{C}}}_{2}$. Then the interpolation constraints are used to construct the interpolation equation of the corresponding implicit function. Finally, we can obtain the implicit function representing the corresponding implicit surface by solving the interpolation equation. More details can be found in the works of the RBF interpolation method.

#### Implicit functions

The implicit function representing the hanging wall surface is defined as ${f}_{1}\left({\varvec{x}}\right)$, the implicit function representing the foot wall surface is defined as ${f}_{2}\left({\varvec{x}}\right)$, the implicit function representing the vein type ore body model is defined as $F\left({\varvec{x}}\right)$, and the implicit function representing the mean surface is defined as ${f}_{0}\left({\varvec{x}}\right)$.

**Figure 3:**
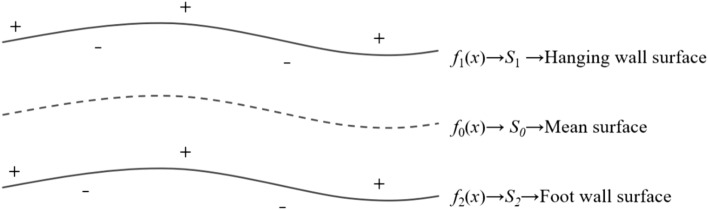
The implicit function fields of the hanging wall surface, the mean surface and the foot wall surface.

1. HW and FW surfaces
  Solve the interpolation equations composed of the interpolation constraints of the hanging wall and foot wall surfaces respectively using the multilevel domain decomposition method^27,28^. Obtain the implicit function ${f}_{1}\left({\varvec{x}}\right)$ that represents the shape trend of the hanging wall surface and the implicit function ${f}_{2}\left({\varvec{x}}\right)$ that represents the shape trend of the foot wall surface. For the solution of the RBF interpolation equation, please refer to the related literatures^29–32^, which will not be repeated here.
2. Mean surface
  The mean surface is a reference surface that fits the medial trend of the hanging wall surface and the foot wall surface. Without loss of generality, we agree that the interior ($F\left({\varvec{x}}\right)<0$) of the vein type geological body model corresponds to the interior (${f}_{1}\left({\varvec{x}}\right)<0$) of the hanging wall surface and the exterior (${f}_{2}\left({\varvec{x}}\right)>0$) of the foot wall surface, as shown in Fig. 3. In this case, the implicit function ${f}_{0}\left({\varvec{x}}\right)$ of the mean surface, representing the trend of the vein surface, can be expressed as
  6 $${f}_{0}\left({\varvec{x}}\right)=0.5\times ({f}_{1}\left({\varvec{x}}\right)+{f}_{2}\left({\varvec{x}}\right))$$
3. Combined vein surface

**Figure 4:**
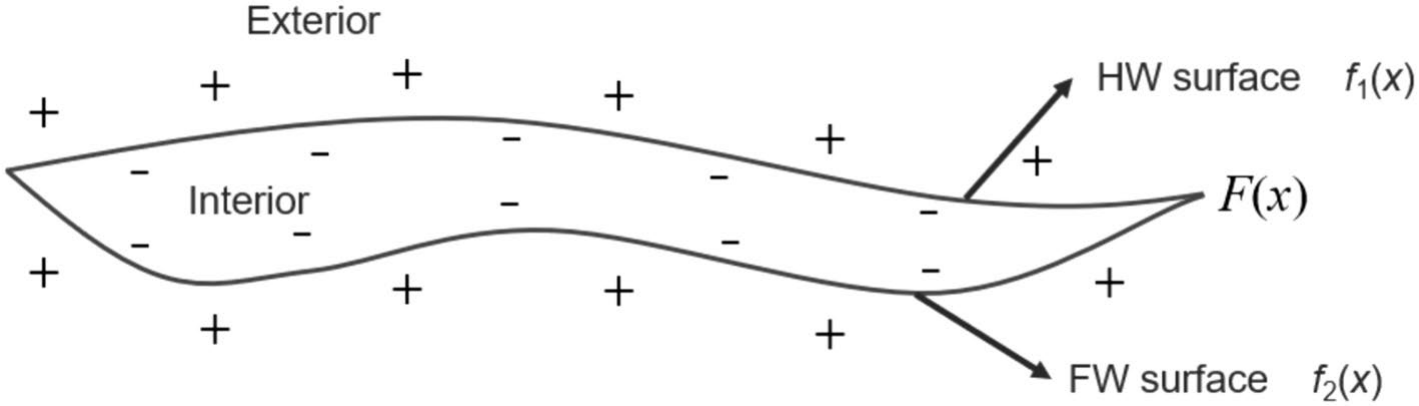
The implicit function field of the combined vein surface.

The vein type ore body model can be seen as the Boolean intersection of the hanging wall surface and the foot wall surface, as shown in Fig. 4. If the hanging wall surface does not intersect with the foot wall surface, a clipping surface should be specified to clip the combined result to form a closed mesh. According to the intersection operation of the Boolean combination constraints, the implicit function $F\left({\varvec{x}}\right)$ representing the vein type ore body model can be expressed as

7 $$F\left({\varvec{x}}\right)={f}_{1}\left({\varvec{x}}\right)\cap \left(-{f}_{2}\left({\varvec{x}}\right)\right)=max\left({f}_{1}\left({\varvec{x}}\right),\left(-{f}_{2}\left({\varvec{x}}\right)\right)\right)$$

In this paper, we mainly focus on the modeling rules between several sub-functions instead of the interpolation process of each sub-function. More details about the implicit modeling method based on the signed distance field and the interpolation of implicit function refer to the relevant references. In practice, the additional interpolation constraints should be constructed to ensure that the vein surface merges or splits along the direction of strike according to the corresponding geological environment.

### Vein thickness constraints

Based on the similar idea of the signed distance field combination, the vein thickness constraints that force the vein to maintain a minimum or maximum thickness can be defined. The basic idea of thickness constraints is to control the thickness of the hanging wall and foot wall surfaces by using combination constraints of the offset mean surface according to a specified thickness.

#### Minimum thickness constraint

To avoid the unexpected holes inside the ore body model, the thickness distribution of the vein object can be adjusted by specifying the minimum thickness constraint.

Define the implicit function ${f}_{10}\left({\varvec{x}}\right)$ of the mean surface offset along the direction of the hanging wall surface according to the specified minimum thickness ${d}_{min}$

8 $${f}_{10}\left({\varvec{x}}\right)={f}_{0}\left({\varvec{x}}\right)-{d}_{min}\times 0.5$$

Define the implicit function ${f}_{20}\left({\varvec{x}}\right)$ of the mean surface offset along the direction of the foot wall surface according to the specified minimum thickness ${d}_{min}$

9 $${f}_{20}\left({\varvec{x}}\right)={f}_{0}\left({\varvec{x}}\right)+{d}_{min}\times 0.5$$

The corrected hanging wall surface constrained by the minimum thickness can be regarded as the union operation of the original hanging wall surface and the offset mean surface. Therefore, the hanging wall surface implicit function ${f}_{1}^{^{\prime}}\left({\varvec{x}}\right)$ constrained by the minimum thickness can be expressed as

10 $${f}_{1}^{^{\prime}}\left({\varvec{x}}\right)={f}_{1}\left({\varvec{x}}\right)\cup {f}_{10}\left({\varvec{x}}\right)=min\left({f}_{1}\left({\varvec{x}}\right),{f}_{10}\left({\varvec{x}}\right)\right)$$

The corrected foot wall surface constrained by the minimum thickness can be regarded as the union operation of the original foot wall surface and the offset mean surface. Therefore, the foot wall surface implicit function ${f}_{2}^{^{\prime}}\left({\varvec{x}}\right)$ constrained by the minimum thickness can be expressed as

10 $${f}_{2}^{^{\prime}}\left({\varvec{x}}\right)={f}_{2}\left({\varvec{x}}\right)\cap {f}_{20}\left({\varvec{x}}\right)=max\left({f}_{2}\left({\varvec{x}}\right),{f}_{20}\left({\varvec{x}}\right)\right)$$

The implicit function ${F}^{^{\prime}}\left({\varvec{x}}\right)$ of the vein type ore body model constrained by the minimum thickness can be expressed as

12 $${F}^{^{\prime}}\left({\varvec{x}}\right)={f}_{1}^{^{\prime}}\left({\varvec{x}}\right)\cap \left(-{f}_{2}^{^{\prime}}\left({\varvec{x}}\right)\right)=max\left({f}_{1}^{^{\prime}}\left({\varvec{x}}\right),\left(-{f}_{2}^{^{\prime}}\left({\varvec{x}}\right)\right)\right)$$

#### Maximum thickness constraint

Similarly, to avoid the unexpected large thickness at local position, the thickness distribution of the vein object can be adjusted by specifying the maximum thickness constraint.

Define the implicit function ${f}_{10}\left({\varvec{x}}\right)$ of the mean surface offset along the direction of the hanging wall surface according to the specified maximum thickness ${d}_{max}$

13 $${f}_{10}\left({\varvec{x}}\right)={f}_{0}\left({\varvec{x}}\right)-{d}_{max}\times 0.5$$

Define the implicit function ${f}_{20}\left({\varvec{x}}\right)$ of the mean surface offset along the direction of the foot wall surface according to the specified maximum thickness ${d}_{max}$

14 $${f}_{20}\left({\varvec{x}}\right)={f}_{0}\left({\varvec{x}}\right)+{d}_{max}\times 0.5$$

The corrected hanging wall surface constrained by the maximum thickness can be regarded as the intersection operation of the original hanging wall surface and the offset mean surface. Therefore, the implicit function ${f}_{1}^{^{\prime}}\left({\varvec{x}}\right)$ of the hanging wall surface constrained by the maximum thickness can be expressed as

15 $${f}_{1}^{^{\prime}}\left({\varvec{x}}\right)={f}_{1}\left({\varvec{x}}\right)\cap {f}_{10}\left({\varvec{x}}\right)=max\left({f}_{1}\left({\varvec{x}}\right),{f}_{10}\left({\varvec{x}}\right)\right)$$

The corrected foot wall surface constrained by the maximum thickness can be regarded as the intersection operation of the original foot wall surface and the offset mean surface. Therefore, the implicit function ${f}_{2}^{^{\prime}}\left({\varvec{x}}\right)$ of the foot wall surface constrained by the maximum thickness can be expressed as

16 $${f}_{2}^{^{\prime}}\left({\varvec{x}}\right)={f}_{2}\left({\varvec{x}}\right)\cup {f}_{20}\left({\varvec{x}}\right)=min\left({f}_{2}\left({\varvec{x}}\right),{f}_{20}\left({\varvec{x}}\right)\right)$$

The implicit function ${F}^{^{\prime}}\left({\varvec{x}}\right)$ of the vein type ore body model constrained by the maximum thickness can be expressed as

17 $${F}^{^{\prime}}\left({\varvec{x}}\right)={f}_{1}^{^{\prime}}\left({\varvec{x}}\right)\cap \left(-{f}_{2}^{^{\prime}}\left({\varvec{x}}\right)\right)=max\left({f}_{1}^{^{\prime}}\left({\varvec{x}}\right),\left(-{f}_{2}^{^{\prime}}\left({\varvec{x}}\right)\right)\right)$$

### Vein boundary constraints

### Boundary clipping constraint

Considering that there is no valid geological sampling data near the ore body boundary, the boundary clipping constraint should be specified to control the vein model boundary. For the vein model with a certain thickness at the boundary, the boundary clipping constraint can ensure that the model cut off at the specific position. There are several ways can be considered to clip the model without considering pinch out.

A very natural way is to clip the implicit function by defining a clipping function and combining the Boolean combination constraints. The clipping function can be obtained by interpolating some manually specified interpolation constraints. The advantage of this method is that it is convenient to construct a better clipping function for the complex vein objects.

Another simpler way is that define a specified oriented and closed polyline as a clipping line to clip the polygon mesh directly. As an available approach, we define the mean plane where the closed polyline is located as the direction of the polyline. It is worth noting that the oriented and closed polyline can be converted into a simple clipping function based on the idea of signed distance field. The implicit function of an oriented and closed polyline ${\varvec{L}}$ can be expressed as

18 $${f}_{3}\left({\varvec{x}}\right)={sign}_{{\varvec{x}}}\times mindist\left({{\varvec{x}}}^{^{\prime}},{{\varvec{L}}}^{^{\prime}}\right)$$

where ${\varvec{x}}$ is a point in the mineralized domain, ${{\varvec{x}}}^{^{\prime}}$ is the projection point of ${\varvec{x}}$ on the mean plane, and ${{\varvec{L}}}^{^{\prime}}$ is the projection line of ${\varvec{L}}$ on the mean plane. $mindist({{\varvec{x}}}^{^{\prime}},{{\varvec{L}}}^{^{\prime}})$ represents the nearest distance between ${{\varvec{x}}}^{^{\prime}}$ and ${{\varvec{L}}}^{^{\prime}}$ on the mean plane. ${sign}_{{\varvec{x}}}$ is the sign of ${\varvec{x}}$ for the corresponding implicit function ${f}_{3}\left({\varvec{x}}\right)$.

Without loss of generality, we agree that the sign of projection point is negative when it is inside the closed polyline and positive when it is outside the closed polyline. In this case, the implicit function ${F}^{^{\prime}}\left({\varvec{x}}\right)$ can be expressed as

19 $${F}^{^{\prime}}\left({\varvec{x}}\right)=F\left({\varvec{x}}\right)\cap {f}_{3}\left({\varvec{x}}\right)=max\left(F\left({\varvec{x}}\right),{f}_{3}\left({\varvec{x}}\right)\right)$$

#### Pinch out constraint

Generally, the pinch out lines obtained by naturally extending the hanging wall and foot wall surfaces to the intersection position are difficult to satisfy the geological rules of mineralization. Therefore, the vein surface should be allowed pinching out at the specific lines specified by the geological engineers. It is useful to provide a convenient pinch out constraint that allows the geological engineers to control the pinch out position of ore body interactively.

A very simple idea is to take the pinch line as the additional interpolation constraints of the hanging wall and foot wall surfaces directly. However, due to the nature of the implicit modeling method the two surfaces may not intersect at the pinch line exactly. To ensure the vein surface pinch out where required, we consider controlling the thickness of the vein surface at a specific position based on the idea of signed distance field. For example, the pinch out constraints can be constructed based on the boundary clipping constraints. The basic idea of this method is to construct the variable thickness constraint by using the implicit function of oriented and closed polyline ${\varvec{L}}$. Compared with the boundary clipping constraint, pinch out constraint has the following characteristics: the thickness of vein surface decreases gradually insider the pinch out line, and the thickness of the vein surface on and outside the pinch out line is equal to zero. Therefore, we consider constructing the variable maximum thickness constraint to limit the vein surface based on the boundary clipping constraint. A simple variable maximum thickness constraint ${D}_{max}$ can be expressed as

20 $${D}_{max}=\left\{\begin{array}{l}\delta \times \left|{f}_{3}\left({\varvec{x}}\right)\right|, \quad {f}_{3}\left({\varvec{x}}\right)<\varepsilon \\ 0, \quad {f}_{3}\left({\varvec{x}}\right)\ge 0\end{array}\right.$$

where $\delta$ is the pinch coefficient of the vein surface, $\varepsilon$ is used to control the starting position of pinch out.

### Surface reconstruction

#### Isosurface extraction

Based on the above defined modeling rules, the combined implicit function representing the complete ore body model is obtained. However, as mentioned earlier, the isosurface of the combined implicit function should not be extracted directly.

1. Reconstruct the HW surface
  Extract the isosurface ${{\varvec{S}}}_{1}$ of the hanging wall surface represented by ${f}_{1}\left({\varvec{x}}\right)$ or ${f}_{1}^{^{\prime}}\left({\varvec{x}}\right)$ using the implicit surface reconstruction method.
2. Reconstruct the FW surface
  Extract the isosurface ${{\varvec{S}}}_{2}$ of the foot wall surface represented by ${f}_{2}\left({\varvec{x}}\right)$ or ${f}_{2}^{^{\prime}}\left({\varvec{x}}\right)$ using the implicit surface reconstruction method.
3. Reconstruct the mean surface
  Extract the isosurface ${{\varvec{S}}}_{0}$ of the mean surface represented by ${f}_{0}\left({\varvec{x}}\right)$ using the implicit surface reconstruction method.
4. Calculate the thickness distribution

To determine the thickness distribution of the vein object in the mineralization domain, the signed thickness field can be defined by the signed distance between the hanging wall and foot wall surfaces, and the corresponding the implicit function $T\left({\varvec{x}}\right)$ can be represented as

21 $$T\left({\varvec{x}}\right)={f}_{2}\left({\varvec{x}}\right)-{f}_{1}\left({\varvec{x}}\right)$$

**Figure 5:**
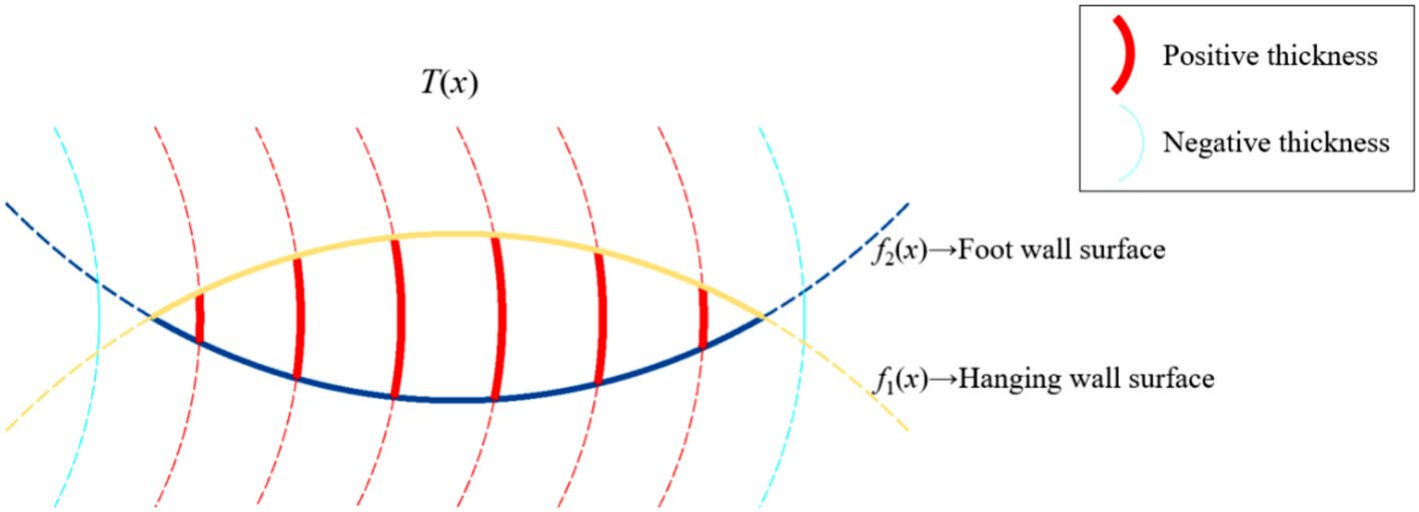
A schematic diagram of the thickness distribution of signed thickness field.

The positive thickness indicates that the hanging wall surface is on the upper side of the foot wall surface, and the negative thickness indicates that the foot wall surface is on the upper side of the hanging wall surface. Therefore, the sign of $T\left({\varvec{x}}\right)$ represents the positional relationship between the hanging wall and foot wall surfaces, which can be used to analyze the thickness distribution of the vein object, as shown in Fig. 5. The thickness value of any point inside the ore body model satisfies $T\left({\varvec{x}}\right)\ge 0$.

#### Surface Boolean combination

**Figure 6:**
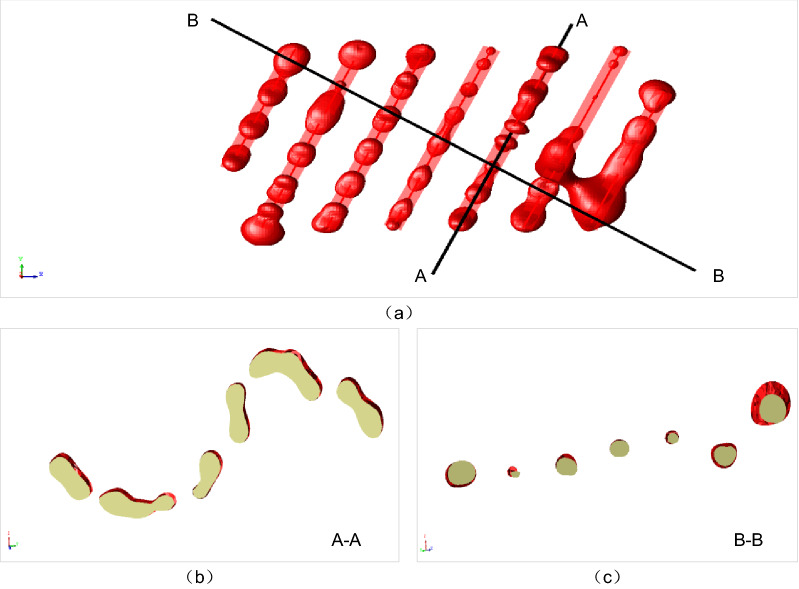
A failure example of the direct interpolation method without separating the HW and FW constraints for a vein type ore body. (**b**,**c**) The details of the reconstructed model (**a**) by cutting along the A-A and B-B planes.

To obtain a complete ore body model, the common approach is to recover the polygon mesh using the isosurface extraction method directly. However, for the vein type ore bodies, the direct reconstruction method is difficult to recover the realistic shape of the corresponding implicit functions with a thin thickness, as shown in Fig. 6.

To avoid the limitation of the direct reconstruction method, an alternative approach is to extract the isosurfaces respectively and combine the results. The method no longer directly extracts the isosurface of the combined implicit function $F\left({\varvec{x}}\right)$ or ${F}^{^{\prime}}\left({\varvec{x}}\right)$, but uses the polygon Boolean operation method to combine the hanging wall and foot wall meshes to form a complete ore body model. The hanging wall and foot wall meshes may be adjusted to snap the pinch out lines. However, if the hanging wall mesh does not intersect with the foot wall mesh, the clipping mesh should be considered to avoid the failure of intersection operation. Additionally, to avoid non-manifold defects in the reconstructed ore body model, it is necessary to check the validity of the polygon mesh and repair the unexpected errors, such as degenerate edges and intersected polygons.

## Experiment results

**Figure 7:**
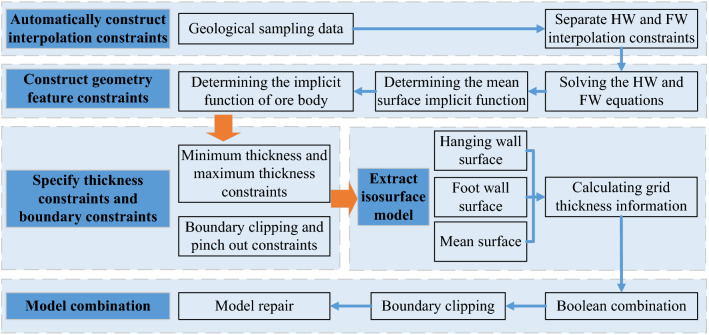
Overall flow chart of the modeling method for narrow vein type ore bodies.

Overall, the automatic modeling process of narrow vein ore body modeling method is shown in Fig. 7. We implemented the vein surface modeling method using the C++ programing language.

To validate the viability of the method, we modeled several narrow vein type ore bodies based on several real geological sampling datasets. In the experiment examples, we used the radial basis functions interpolant as the implicit function to interpolate the implicit surface. More details about the existing implicit geological modeling method can refer to some pioneer works^1,5,33^.

**Figure 8:**
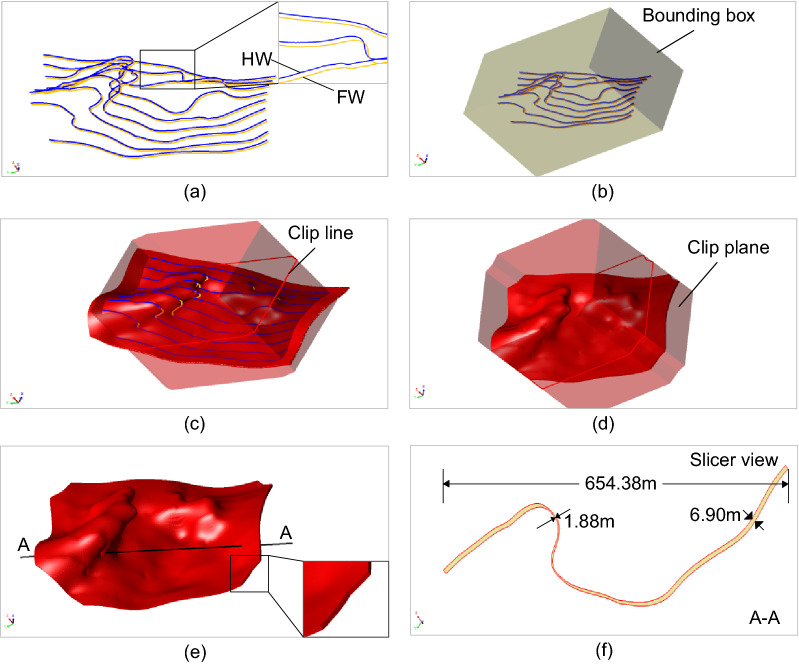
Implicit modeling of a narrow vein orebody model. (**a**) Contour polyline data; (**b**) rectangle bounding box; (**c**) initial model; (**d**) clipped model; (**e**) final model; (**f**) slicer view.

**Figure 9:**
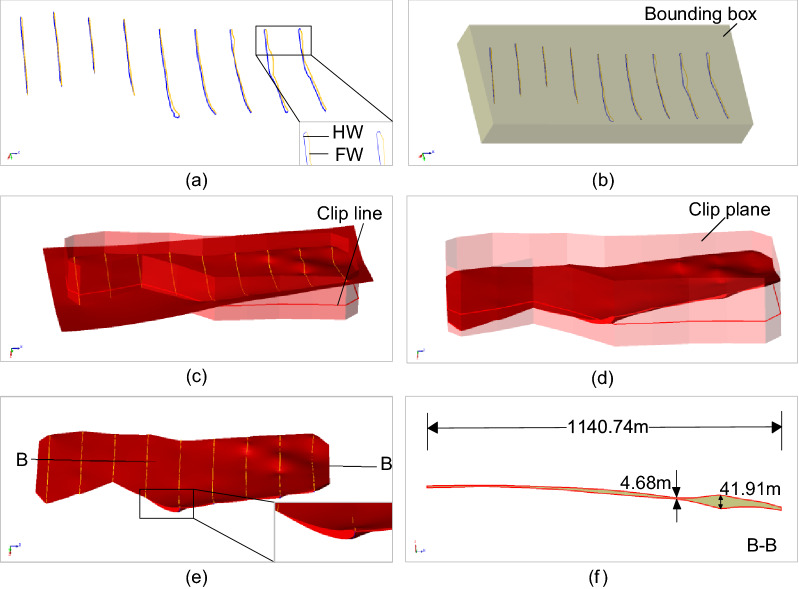
Implicit modeling of a narrow vein orebody model. (**a**) Contour polyline data; (**b**) rectangle bounding box; (**c**) initial model; (**d**) clipped model; (**e**) final model; (**f**) slicer view.

**Figure 10:**
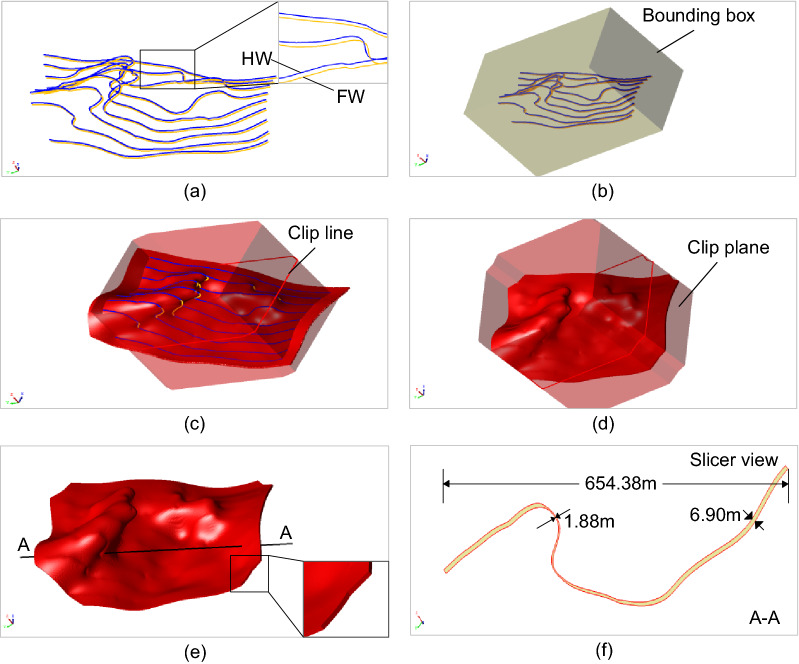
Implicit modeling of a narrow vein orebody model. (**a**) Contour polyline data; (**b**) rectangle bounding box; (**c**) initial model; (**d**) clipped model; (**e**) final model; (**f**) slicer view.

**Figure 11:**
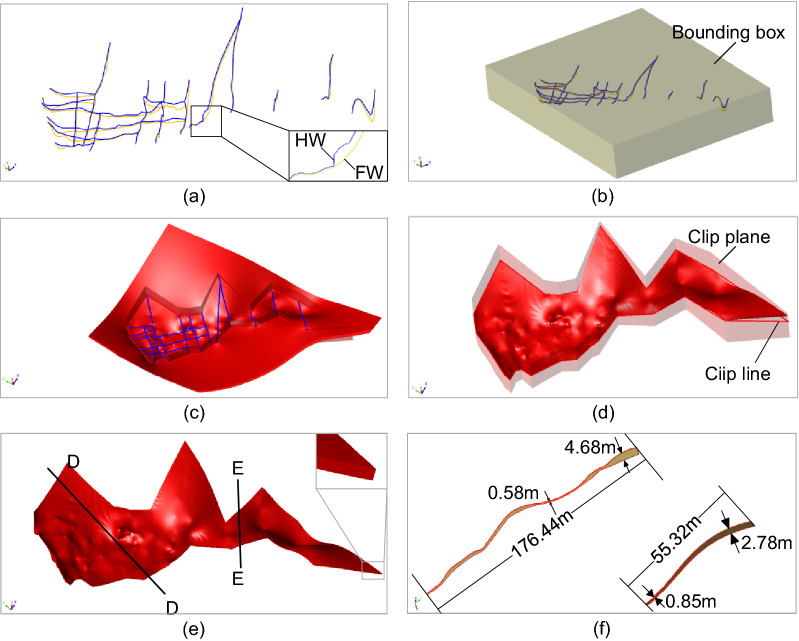
Implicit modeling of a narrow vein orebody model. (**a**) Contour polyline data; (**b**) rectangle bounding box; (**c**) initial model; (**d**) clipped model; (**e**) final model; (**f**) slicer view.

**Figure 12:**
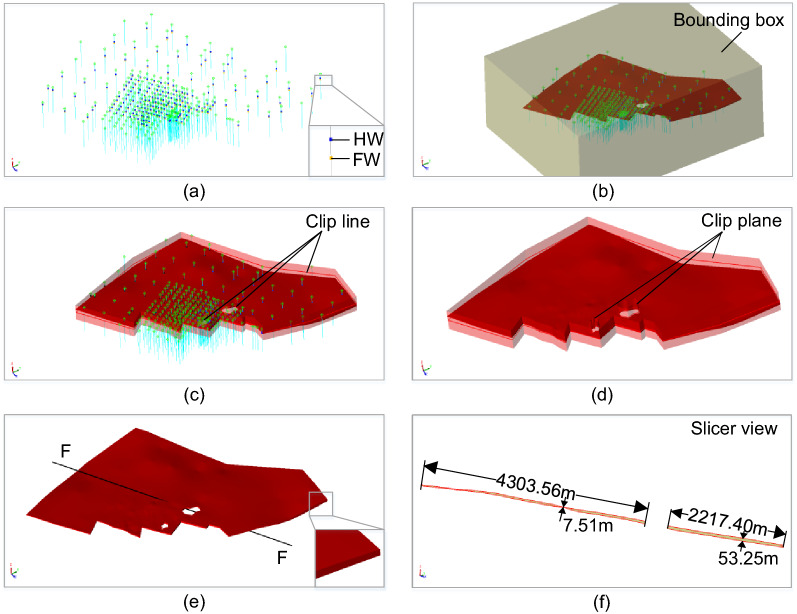
Implicit modeling of a narrow vein orebody model. (**a**) Drillhole data; (**b**) rectangle bounding box; (**c**) initial model; (**d**) clipped model; (**e**) final model; (**f**) slicer view.

Several real geological data sets are used to demonstrate the modeling effects for narrow vein type ore bodies, as shown in Figs. 8, 9, 10, 11 and 12. The hanging wall and foot wall surfaces are interpolated by the interpreted contour polyline data (Figs. 8, 9, 10, 11) and the drillhole data (Fig. 12) using the implicit modeling method. The complete ore body models are obtained by combining the hanging wall and foot wall surfaces using the polygon Boolean combination operation method.

Figure 8 shows an example of the process of the vein surface modeling method. First, the contour polylines interpreted from the geological sampling data are divided into the hanging wall and foot wall polylines. The polylines are converted into the interpolation constraints. The rectangle bounding box in Fig. 8b represents the mineralization domain of the vein object. As the hanging wall surface extends to the rectangle bounding box and does not intersect with the foot wall surface, an oriented and closed polyline (Fig. 8c) is defined to clip the vein boundary. The final vein object (Fig. 8e) is obtained by combing the hanging wall and foot wall surfaces.

The modeling results show that the vein surface modeling method can be used to recover the narrow vein structure of geological bodies effectively. For example, the maximum length of the vein model in Fig. 11 can be several hundreds of meters long. The thickness of the vein model in Fig. 8 ranges from 0 to 10 m, and the average thickness is about 0.5–1.5 m (Fig. 11f). For the model in Fig. 11, to avoid internal voids, a minimum thickness constraint is specified to ensure that the hanging wall surface does not intersect with the foot wall surface. It is worth noting that voids may occur naturally if there are no drillhole data, which should be determined according to the actual geological conditions. The pinch out constraint combined with a clipping polyline is used to control the boundary of the vein surface. Additionally, the hanging wall and foot wall meshes are adjusted to snap the pinch out lines exactly. The final vein object (Fig. 11e) is obtained by combing the hanging wall and foot wall surfaces.

## Conclusion and discussion

In this paper, we consider improving the automation and modeling effect of orebody implicit modeling by automatically constructing modeling rule constraints that satisfy the geometry shape features of specific type of ore bodies. Taking the narrow vein type models as an example, we implement an implicit modeling method of narrow vein type ore bodies using the modeling constraints considering the geometry shape features of geological body. The method can recover the geometry shape features of the realistic models well. It improves the automation of implicit modeling and the modeling effect for vein type ore bodies, which ensures that the modeling results honor the priori geological knowledge well. In addition, it is clear that the method can be also applied to similar structures like narrow dykes, lenses and seams which are hard to be modelled directly by the direct interpolation methods. Taking the implicit function of radial basis functions interpolation as an example, several experiments are carried out by using the real geological sampling data of the mines. The experimental results show that the method is suitable for the modeling of narrow vein type ore bodies.

To solve the problem of geological body modeling based on sparse data interpolation, we discuss the implicit modeling method considering specific geological rules, especially the constraints of geometry shape features. The vein type surface modeling method provides an available idea to extend the constraint types of implicit modeling from the perspective of modeling rules. In general, the implicit modeling idea of constructing modeling rules based on geometry shape features is conducive to controlling the global shape features of the model. For the vein type models, the minimum thickness constraint can be used to ensure that the hanging wall and foot wall surfaces do not cross, and the maximum thickness constraint can be used to avoid over extrapolation of the vein surface model. The pinch out constraint can be combined with the minimum thickness constraint to ensure that there is no gap inside the vein model that honors geological sampling data. It is an important task to improve the reliability and automation of the implicit modeling method. Based on the similar idea, we consider developing more convenient modeling rules in the future.

For the vein type surface modeling, there are still some modeling rules that should be further improved, including the optimization of thickness of the model and the control of model extrapolation. In terms of thickness optimization, besides the rough control of minimum and maximum thickness constraints, we consider optimizing the thickness distribution of the model through the uncertainty analysis of the thickness field under the condition of satisfying structural geological conditions. A feasible way is to analyze the uncertainty of the thickness of the vein model by using the theory of geostatistics in combination with the thickness trend of the geological body. It is worth noting that when the thickness value of the maximum thickness constraint is less than the thickness value of the minimum thickness constraint, the constraint conditions will conflict and affect the actual thickness constraint effect. Moreover, if the thickness of the surface tends to zero, it is also a major challenge for the reconstruction method. In terms of model extrapolation, it is necessary to develop additional constraints that can flexibly control the position of pinch out and limit the distribution trend of model extrapolation in areas without data support. For example, the protrusions in the experiment results are caused by the interpolation without considering geological anisotropic trends. It is a common challenge to determine the pinch-out boundary for complex ore bodies. For the ore body modeling, it is often necessary to make the vein surface pinch out at the specified lines automatically according to the inferred geological trends. And further study is needed to improve the reliability and efficiency of narrow vein surface modeling in the future. We mainly discuss the interpolation of vein type surface, while the isosurface extraction of vein type surfaces should be further studied. For example, the problems of reconstructing the sharp features of the implicit surface and snapping the given geological sampling data (especially the contour polylines) in the process of reconstruction should be further studied.

## Data Availability

All experiment data could be made available to qualified investigators upon reasonable request by contacting the corresponding author (L.B.).
